# An Ultraviolet Sensor and Indicator Module Based on p–i–n Photodiodes

**DOI:** 10.3390/s19224938

**Published:** 2019-11-13

**Authors:** Yu-Chieh Chiu, Pinghui Sophia Yeh, Tzu-Hsun Wang, Tzu-Chieh Chou, Cheng-You Wu, Jia-Jun Zhang

**Affiliations:** 1 Department of Electronic and Computer Engineering, National Taiwan University of Science and Technology, Taipei 106, Taiwan; 2 Graduate Institute of Electro-Optical Engineering, National Taiwan University of Science and Technology, Taipei 106, Taiwan

**Keywords:** UV sensors, light-emitting diodes, photodiodes, indium-tin oxide

## Abstract

The monolithic integration of an ultraviolet (UV) sensor and warning lamp would reduce the cost, volume, and footprint, in comparison to a hybrid combination of discrete components. We constructed a module comprising a monolithic sensor indicator device based on basic p–i–n (PIN) photodiodes and a transimpedance amplifier. GaN-based light-emitting diodes (LEDs) with an indium-tin oxide (ITO) current-spreading layer and PIN photodiodes without ITO deposition on the light-receiving area, were simultaneously fabricated. The resultant incident photon-to-electron conversion efficiencies of the PIN photodiodes at UV wavelengths were significantly higher than those of the reverse-biased LEDs. The photocurrent signals of the PIN photodiode were then converted to voltage signals to drive an integrated visible LED, which functioned as an indicator. The more the ambient UV-light intensity exceeded a specified level, the brighter the glow of the LED. The responsivities of 0.20 and 0.16 A/W were obtained at 381 and 350 nm, respectively, under a bias voltage of 5 V. We also addressed the epitaxial structural details that can affect the collection efficiency of the photocurrent generated by UV light absorption. The crosstalk between the PIN photodiode and LEDs (of various center-to-center distances) was measured.

## 1. Introduction

Ultraviolet (UV) light detection has become increasingly important. In particular, UV light-emitting diodes (LEDs) have been applied in various fields, such as epoxy curing, air and water sterilization, and surface disinfection. Appropriate UV light monitoring is essential to protect people from UV damage. UV sensors can also be used in flame detection, biomedical spectroscopy, and military applications [1,2]. III-nitride material has a suitable large bandgap energy, high quantum efficiency and high electron mobility. Many types of AlGaInN-based photodetectors (PDs) have been fabricated and tested [3,4,5,6,7,8,9,10,11,12,13,14,15,16,17,18,19,20,21,22,23,24,25,26,27,28]. Schottky barrier PDs have been commercialized by several companies, and the typical responsivities of this type of PD are 0.18, 0.13, and 0.06 A/W at 350, 300, and 254 nm, respectively [3]. The typical GaN p–i–n (PIN) PD has a peak responsivity of approximately 0.2 A/W in the UV-A wavelength range [4,5,6]. High-responsivity PDs (e.g., avalanche photodiodes, heterojunction phototransistors and photoconductors) generally require complex epitaxial structures or compromises in the response speed. Jiang et al. reported the monolithic integration of LEDs and Schottky barrier PDs for bi-directional optical wireless communication [29]. Li et al. reported the monolithic integration of photodiodes, LEDs, and waveguides for applications in visible light communication [30,31]. Wang et al. conducted in-plane data transmission among a transmitter (LED), waveguide, and receiver (photodiode) [32,33]. Liu et al. reported the monolithic integration of high electron mobility transistors, LEDs, and two types of photodiodes by selective-area epitaxy [34]. In this study, we propose and test a new application of a monolithic LED and PIN photodiode. UV light was detected with a PIN photodiode that sent signals to drive an integrated LED, which served as a warning lamp. We fabricated LEDs and PIN photodiodes with and without indium-tin oxide (ITO) deposition on the light-emitting and receiving area, respectively, in order to avoid UV light absorption by the ITO [14,35]. A sensor indicator module was then developed.

## 2. Device Design and Processing

**Figure 1:**
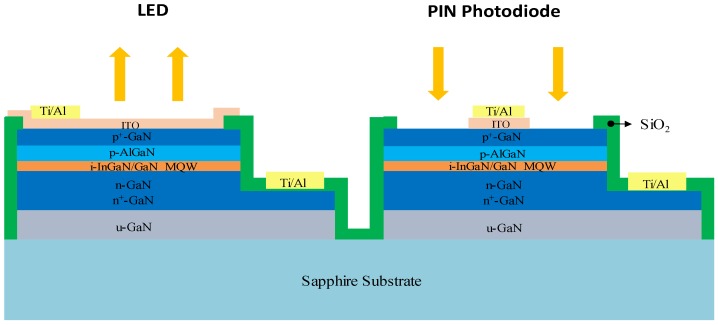
Schematic cross section of the monolithic light-emitting diode (LED) and p–i–n (PIN) photodiode.

**Figure 2:**
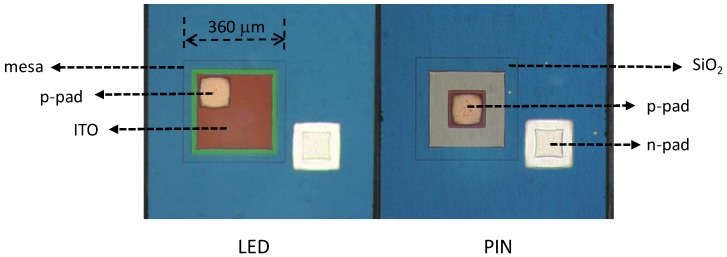
A top-view photo of the monolithic LED and PIN photodiode.

The device structure of the PIN photodiode and LED is illustrated in Figure 1. We used a commercial LED wafer, which had a photoluminescence peak wavelength of approximately 437 nm and a full-width-at-half-maximum of 22 nm. The wafer was grown on a c-plane patterned sapphire substrate. From top to bottom, the epitaxial structure comprised a 100-nm p-GaN layer, a 100-nm p-Al_0.2_Ga_0.8_N layer (doping concentration = 3 × 10^17^ cm^−3^), 15-pair In_0.12_Ga_0.88_N/GaN multiple quantum wells (total thickness = 210 nm), a 100-nm n-GaN layer, and a few-micron n^+^-GaN layer. For the efficient collection of photocurrent, the p-electrode of the photodiode was located at the center of the device. The processing procedure is described as follows. Each device was isolated by a deep etch that extended down to the insulating sapphire substrate through inductively coupled plasma reactive-ion etching (ICP-RIE). Next, ICP-RIE was used to produce mesas on the LED and photodiode, which provided access to the n-GaN layer. Subsequently, a SiO_2_ insulation/passivation layer was deposited. Next, an electron beam evaporator was used to deposit an ITO layer with a thickness of approximately 200 nm and a composition of 5 wt% SnO_2_ and 95 wt% In_2_O_3_. The ITO layer was then thermally annealed at 625 °C in ambient N_2_ for 8 min; the two functions of the ITO were current spreading for LEDs and ohmic p-contact for both devices. Finally, p/n-metal electrodes were formed. Figure 2 shows a photo of the fabricated PIN photodiode and LED.

## 3. Device Characterization

**Figure 3:**
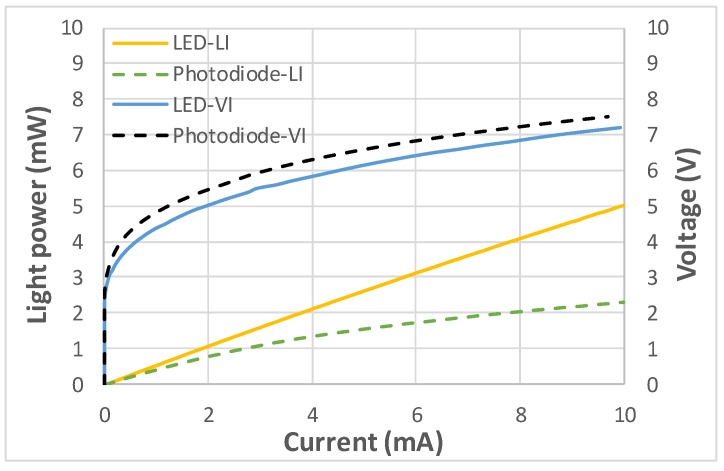
Light–current–voltage (L–I–V) characteristics of the LED and forward-biased photodiode.

**Figure 4:**
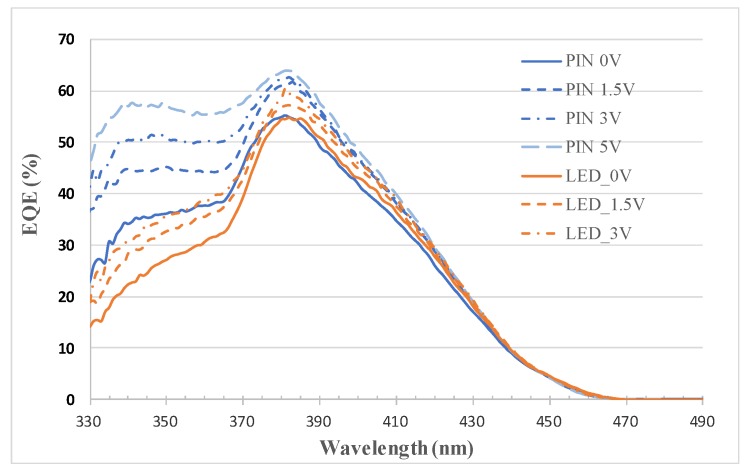
External quantum efficiency (EQE) versus wavelength of the photodiode and reverse-biased LED at various bias voltages.

Figure 3 illustrates the light–current–voltage (L–I–V) characteristics of the LED and forward-biased photodiode. Both the LED and photodiode had an effective area of 0.12 mm^2^, which is equivalent to the mesa area of 360 μm × 360 μm subtracted by the size of the p-metal pad, that is, 100 μm × 100 μm. Compared with the LED, the photodiode had a significantly lower slope efficiency—which is defined as the quotient of the optical output power and injection current—in addition to a marginally higher forward voltage and nearly equal series resistance. These photodiode characteristics are attributable to the considerably smaller ITO layer on the photodiode than on the LED. This smaller ITO layer decreased current-spreading area, which resulted in a decreased light-emitting area and marginally increased total resistance. Moreover, the external quantum efficiency (EQE) of the photodiode and reverse-biased LED were measured by employing an incident photon-to-electron conversion efficiency (IPCE) apparatus, which comprised a broadband 300-W Xenon lamp (Newport, model #6258), monochromator (Newport, model #74024), chopper, and lock-in amplifier (Newport, SR830). The results are plotted in Figure 4. The EQE of the photodiode was higher than that of the reverse-biased LED, especially at wavelengths shorter than 365 nm and at high bias voltages. This result is attributable to the following reasons. First, the ITO layer in general has low transmittance at UV wavelengths [14,35]. Therefore, the ratio of the EQE at 350 nm to the EQE at 381 nm increased from 50% (with 200-nm-thick ITO) to 65% (without ITO) at zero bias. Second, short-wavelength light has a relatively high absorption coefficient (and by implication, a relatively short absorption length). Thus, the EQE value of the short-wavelength light was more sensitive to increased bias voltage, which produced a wider depleted region with one edge closer to the illuminated surface. The increase in the depletion width in relation to the bias voltage depends on the doping level of the p-cladding layer, and its effectiveness in improving the EQE value, also depends on the thickness of the p-GaN layer. For example, PIN photodiodes made of another epitaxial wafer having a 200-nm-thick p-GaN layer did not exhibit significant increase in EQE at short wavelengths under increased bias voltage. Because GaN absorbed wavelengths shorter than 365 nm and produced electron-hole pairs, the thick p-GaN layer caused electron-hole pair recombination before separation by the field of the depleted region. Moreover, both devices exhibited a saturation in EQE at high bias voltages, and their cut-off wavelengths were approximately 460 nm, as expected. At 381 nm, the peak responsivity of the photodiode at 0 and 5 V was approximately 0.17 and 0.20 A/W, respectively. At 350 nm, the responsivity was approximately 0.10 and 0.16 A/W at 0 and 5 V, respectively. The EQE at wavelengths shorter than 330 nm could not be measured because of a lack of incident light from the Xenon lamp.

## 4. Module Design and Results

**Figure 5:**
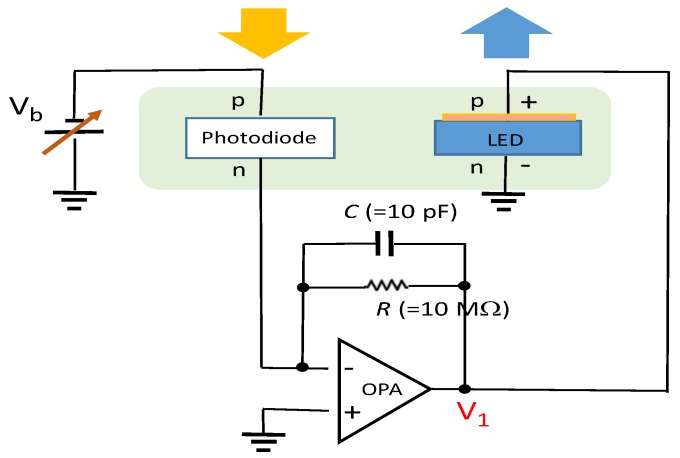
Schematic diagram of the UV sensor and indicator module comprising a PIN photodiode, transimpedance amplifier, and visible LED, where OPA represents the operational amplifier.

**Figure 6:**
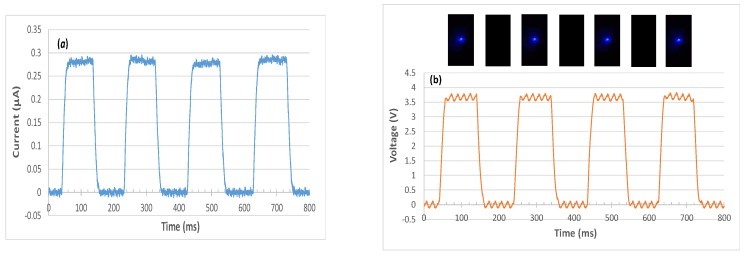
(**a**) Temporal response of the photocurrent detected by the PIN photodiode at 0-V bias, and (**b**) the corresponding output voltage V_1_ of the transimpedance amplifier, to a 5-Hz optical pulse signal at 389 nm with 60 Hz alternating current noise. The inset photos exhibit the integrated LED being switched on and off accordingly.

**Figure 7:**
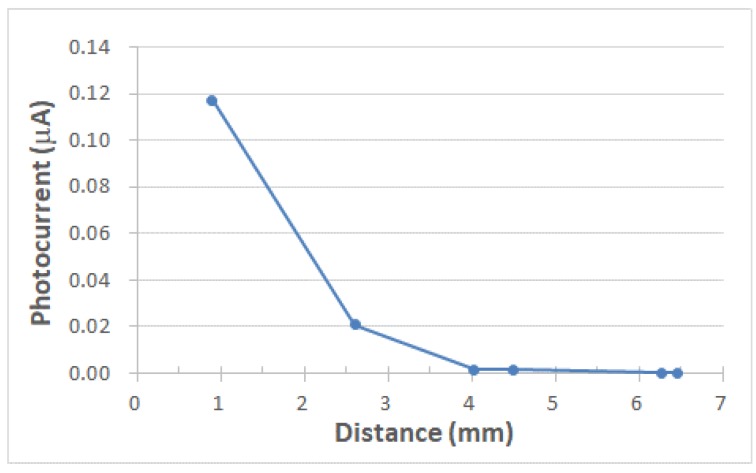
Photocurrent versus center-to-center distance between the PIN photodiode and LED. The surface-emitting power of the LED was 1 mW.

We built a sensor indicator module that detects UV light through a PIN photodiode. Specifically, the photodiode converts photocurrent signals to voltage signals through an external transimpedance amplifier, and the resulting voltage signals are used to drive an integrated LED as the indicator. The schematic of the designed module is displayed in Figure 5. The transimpedance amplifier was a typical operational amplifier connected in the inverting amplifier configuration with negative feedback. The current to voltage gain equals the feedback resistance *R* at low frequencies. The circuit bandwidth in this design was approximately 1.6 kHz, which was considerably larger than the current operating frequency of approximately 5 Hz. Specifically, we operated the module at such a low frequency that we could visually inspect the on-off switch of the LED despite the persistence of vision. The response speed of a PIN photodiode is of the order of nanoseconds [30]. The resistance (*R*) value was configured to be such that the LED indicator was switched on once a specified UV light intensity was exceeded. Assuming that the specified UV intensity level was 1.5 mW/cm^2^ at 389 nm, the associated illuminating current at a UV intensity of 1.7 mW/cm^2^ should, in theory, be converted to an output voltage (V_1_) larger than the turn-on voltage of the LED. Figure 6 illustrates the resultant photocurrent and voltage signals associated with a 5-Hz optical pulse signal at 389 nm. The integrated LED was switched on and off accordingly. In addition, we noticed crosstalk between the photodiode and LED when the distance between them was too small or when the LED power was large. Light emission from an LED can be partially coupled into neighboring photodiodes through a patterned sapphire substrate. The degree of crosstalk was evaluated by measuring, in a dark environment, the photocurrent of the PIN photodiode when only one LED was lit. Figure 7 illustrates the measured photocurrent in relation to the center-to-center distance between the photodiode and LED when the photodiode was biased at 0 V and the surface-emitting power of the LED was a continuous wave at 1 mW. When using an LED with a power level less than 1 mW and a distance larger than 4 mm, the crosstalk can be reduced to negligible levels. A light deviating design can be added in future modifications to minimize crosstalk. Furthermore, if an LED wafer with a peak wavelength in the violet band (e.g., at 405 nm) is used, the cut-off wavelength of the PIN photodiode would considerably decrease at the expense of the luminous intensity of the LED.

## 5. Conclusions

A GaN-based UV sensor and indicator module comprising an integrated PIN photodiode and LED was designed and tested. For UV detection, photocurrent signals from UV light were converted to voltage signals to drive an integrated LED (functioning as a warning lamp). The output power can be scaled up by using multiple or large LEDs when crosstalk is contained. Responsivity in the UV range was improved by (1) having a thin GaN cap layer, (2) having low doping in the p-cladding layer, and (3) not having ITO deposition on the light-receiving area. The responsivities of 0.20 and 0.16 A/W were obtained at 381 and 350 nm, respectively, under a bias voltage of 5 V. The designed device could be valuable to LED manufacturers.
